# Parliament and the Profession

**Published:** 1919-07-26

**Authors:** 


### Parliament and the Profession.

### Alleged Insubordination at a Military Hospital.

Sir Stuart Coats asked if the Secretary of State for War was aware that the military hospital at Warlingham is understaffed and the supervision of the men inadequate, thefts being frequent and patients breaking bounds whenever they wish, cutting the barbed wire and violently assaulting those who try to restrain them, afterwards wandering to Croydon and other places, the general effect being that the discipline of the soldiers on guard is much impaired. Mr. Churchill said that inquiries into the statement are being made.

### Transference of Military Patients.

Mr. Winston Churchill, in answering a question put by Capt. W. Benn, admitted that complaints had been received as to the position and suitability of Paddington Military Hospital, a converted workhouse, which had received cases evacuated at King George Hospital, and stated that a more suitable building was being looked for.

Major Glyn asked as to the position of officers and men in military hospitals which are to be taken over by the Ministry of Pensions on August 1, and was assured by Mr. Forster that no transfer will take place when injury to the patient's health would be likely to be caused thereby.

